# A Framework to Assess the Impact of New Animal Management Technologies on Welfare: A Case Study of Virtual Fencing

**DOI:** 10.3389/fvets.2018.00187

**Published:** 2018-08-21

**Authors:** Caroline Lee, Ian G. Colditz, Dana L. M. Campbell

**Affiliations:** ^1^CSIRO, Agriculture and Food, FD McMaster Laboratory, Armidale, NSW, Australia; ^2^Adjunct to School of Environmental and Rural Science, University of New England, Armidale, NSW, Australia

**Keywords:** animal welfare, cattle, cognition, cognitive activation theory of stress, sheep

## Abstract

To be ethically acceptable, new husbandry technologies and livestock management systems must maintain or improve animal welfare. To achieve this goal, the design and implementation of new technologies need to harness and complement the learning abilities of animals. Here, from literature on the cognitive activation theory of stress (CATS), we develop a framework to assess welfare outcomes in terms of the animal's affective state and its learned ability to predict and control engagement with the environment, including, for example, new technologies. In CATS, animals' perception of their situation occurs through cognitive evaluation of predictability and controllability (P/C) that influence learning and stress responses. Stress responses result when animals are not able to predict or control both positive and negative events. A case study of virtual fencing involving avoidance learning is described. Successful learning occurs when the animal perceives cues to be predictable (audio warning always precedes a shock) and controllable (operant response to the audio cue prevents receiving the shock) and an acceptable welfare outcome ensues. However, if animals are unable to learn the association between the audio and shock cues, the situation retains low P/C leading to states of helplessness or hopelessness, with serious implications for animal welfare. We propose a framework for determining welfare outcomes and highlight examples of how animals' cognitive evaluation of their environment and their ability to learn relates to stress responses. New technologies or systems should ensure that predictability and controllability are not at low levels and that operant tasks align with learning abilities to provide optimal animal welfare outcomes.

## Introduction

The development of new husbandry systems and management technologies has increased the complexity of the environment farmed animals must learn to engage with. For example, cattle may need to learn how to interact with automated milking systems and virtual fences, and laying hens need to learn temporal and spatial design features of new free range and aviary systems. Cognitive and learning abilities vary between individuals and contribute to fitness and survival within wild populations (1, 2). For farmed animals, variation in learning ability (speed to learn and ability to master new tasks) may influence the impact of new technologies on individuals and thus have welfare consequences for introduction of new livestock management systems.

A theory proposed by Ursin and Eriksen (3), termed the cognitive activation theory of stress (CATS), describes concepts that are relevant when considering how animals learn to interact with new farming technologies and systems. The CATS describes the relationship between cognitive evaluation (appraisal) and the stress responses based on studies in rats and humans. Specifically, the stress response relates to what the animal has learned to expect in response to a stimulus. Whether a stimulus is positive or negative depends on the individual appraisal of the situation, which is based on previous experience and expectations of the outcomes of stimuli. Expectancy occurs when the animal registers, stores, and uses information about what stimulus precedes a following stimulus (learning). Ursin and Eriksen (3) describe two stages of learning. The first is termed classical conditioning and is stimulus-stimulus learning involving acquisition of stimulus expectancies, and the second is operant conditioning involving acquisition of response expectancies. The CATS is an activation theory as the stimuli may induce arousal that is indicated through a measurable stress response, such as activation of the hypothalamic pituitary adrenal axis. In general, the welfare outcomes of the two stages of learning are higher for positive expectations (e.g., positive reinforcement) and lower for negative or uncertain expectations (e.g., negative reinforcement) (4, 5).

Both stress and welfare are linked and depend on how an animal perceives its environment (6). Cognitive evaluation of the predictability and controllability (P/C) of a situation are important elements that influence how animals learn and determine whether welfare outcomes will be positive or negative. A classic study in rats conducted by Weiss (7) demonstrates how a lack of P/C can induce stress responses and impact health. When individual rats were placed in identical cages where treatments A and B received identical electric shocks but treatment A rats received a light signal to indicate when a shock was forthcoming (i.e., the shocks were predictable), the stress response in the A rats was similar to controls that did not receive any electric shocks. The B rats displayed high stress responses as evidenced by high corticosterone levels and stomach wall lesions. This same response was seen when A rats were able to prevent an electric shock by turning a wheel (i.e., the shocks were controllable). Surprisingly, the ability to either control or predict the occurrence of the electric shock was equally effective at reducing the stress response, which was explained by the fact that the animals knew they were experiencing a safe period if they hadn't received a warning signal (7).

Bringing together the concepts described above, this paper will propose a framework for determining welfare outcomes based on two dimensions: (1) affective state and (2) predictability and controllability (P/C). We will highlight examples of how animals' cognitive evaluation of their environment and their ability to learn relates to stress responses and animal welfare.

## Predictability and controllability and animal welfare

**Figure 1:**
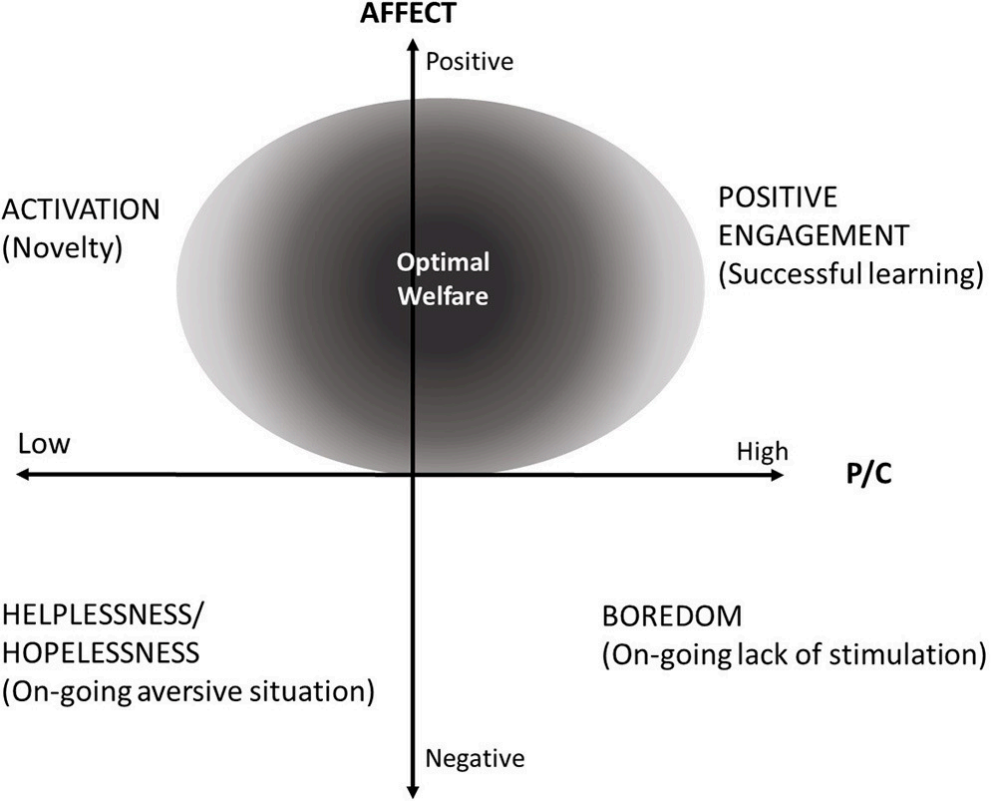
Proposed framework showing the interaction between predictability/controllability (P/C), affect and welfare states. Shaded area indicates intermediate P/C where optimal animal welfare occurs.

The framework (Figure 1) describes the relationship between affective state (as a continuum between positive and negative) and predictability and controllability (low to high). Affect is a core psychological state that modulates neural, behavioral, physiological, and immune functions and hence influences health and productivity (8). For simplicity, we do not decompose affect into its components of valence and arousal. Similarly, we do not separate predictability and controllability in our framework, however, animals may experience situations of high controllability and low predictability and vice versa (9). Cognitive evaluation of environmental characteristics such as predictability and controllability have been demonstrated to trigger emotions in animals (10). Appraisal of other criteria, including suddenness, novelty, and pleasantness have been proposed by Desire et al. (9) as an indicator of emotions in animals. The previously described classical experiments performed on rats by Weiss (7) provide a clear example of how cognitive elements influence stress responses based on predictability and controllability. This has also been demonstrated in livestock species, including sheep where exposure to aversive events that occurred unpredictably and uncontrollably induced chronic stress and negative affective states (11, 12).

Each quadrant of the framework describes an example of a welfare state as a result of the level of P/C and affective states. The examples discussed in relation to the framework do not account for all possible states in each quadrant and as with all frameworks, there are limitations to its use. Not all situations of high predictability and controllability result in good welfare outcomes (see bottom right quadrant; Figure 1). An example is barren environments that are generally highly predictable and not always controllable with animals experiencing negative affective states such as boredom from a lack of stimulation on a daily basis. Barren environments can result in chronic stress as demonstrated by increased ACTH-induced cortisol responses in tethered pigs (13) and stereotypies may result from the animals attempting to cope with the lack of environmental stimulation. There is potential to enhance welfare of animals kept in barren environments by signaling the arrival of a food reward that would increase P/C and improve affective states thereby moving welfare states to the top right quadrant of Figure 1. This presents potential for welfare improvement in intensive systems that may not offer the opportunity to perform a full range of natural behaviors. The top left quadrant is termed novelty. An example of low P/C and positive affect is shown in captive orange-winged Amazon parrots where the frequent rotation of enrichment objects reduced neophobia when compared to provision of new enrichments alone (14). However, individual differences in fearfulness may influence the affective state resulting from this procedure and could create for some individuals a negative welfare outcome.

Even positive events have the ability to compromise welfare if they are not controllable or predictable, for example removal of control by lambs over food delivery induced stress (15). Failure to reward pigs that were taught to perform an operant task to obtain a food reward induced frustration and aggressive behavior (16). In comparison, providing predictability of a food reward enhanced positive emotions as demonstrated in rats (17). This is further supported by evidence that the announcement of the arrival of enrichment (access to a hallway containing mixed grains) to pigs induced more positive emotions than providing enrichment alone (18). The importance of controllability for positive events was demonstrated by pigs that received cognitive enrichment (learning an operant task to access a food reward) displaying positive emotions (less fearful and more exploratory and lower sympathetic activation during feeding), compared to pigs that did not experience cognitive enrichment (19). In these examples, provision of predictability over the arrival of the positive event provides an improved welfare state and animals would move into the top right quadrant of the framework. We have termed this quadrant “Positive engagement” and this aligns with concepts described by Mellor (20). Wechsler and Lea (21) highlight the opportunity to develop enrichment tasks that take into account the learning abilities of animals that may result in improved welfare. Predictable scheduling of feeding in broiler breeder pullets was associated with improved welfare (22). Learning in itself can be motivating and induce emotional responses in animals, for example, heifers showed increased heart rate and more vigorous movement down a race when they made improvements in their learning (23). It has been suggested that a capacity to acquire prediction and control over the environment through learning contributes to resilience of the animal to environmental change (24). Together, evidence of inducing positive affect by providing opportunities for animals to be able to predict and control positive experiences shows much promise for improving welfare of animals kept in confined conditions.

Situations where an individual is exposed to unpredictable and uncontrollable negative events occur when there is an acquired expectancy that no relationships exist between responses and reinforcement (Figure 1: bottom left quadrant). In this situation, the individual perceives that there is no relationship between any action they can do and the outcome and this state is termed helplessness. Hopelessness is similar but with the learned expectancy that all responses lead to a negative result. States of helplessness and hopelessness occur when the animal is not coping and may lead to somatic disease through sustained arousal (3), which has serious implications for animal welfare. Overall, for welfare to be optimal in livestock farming systems, predictability and controllability should not be too high or too low, i.e., it should be at an intermediate level (see circle area in Figure 1). This represents a level that provides stimulation and prevents boredom through providing opportunities to learn that are within the animals cognitive ability.

## A case study: avoidance learning in virtual fencing

**Figure 2:**
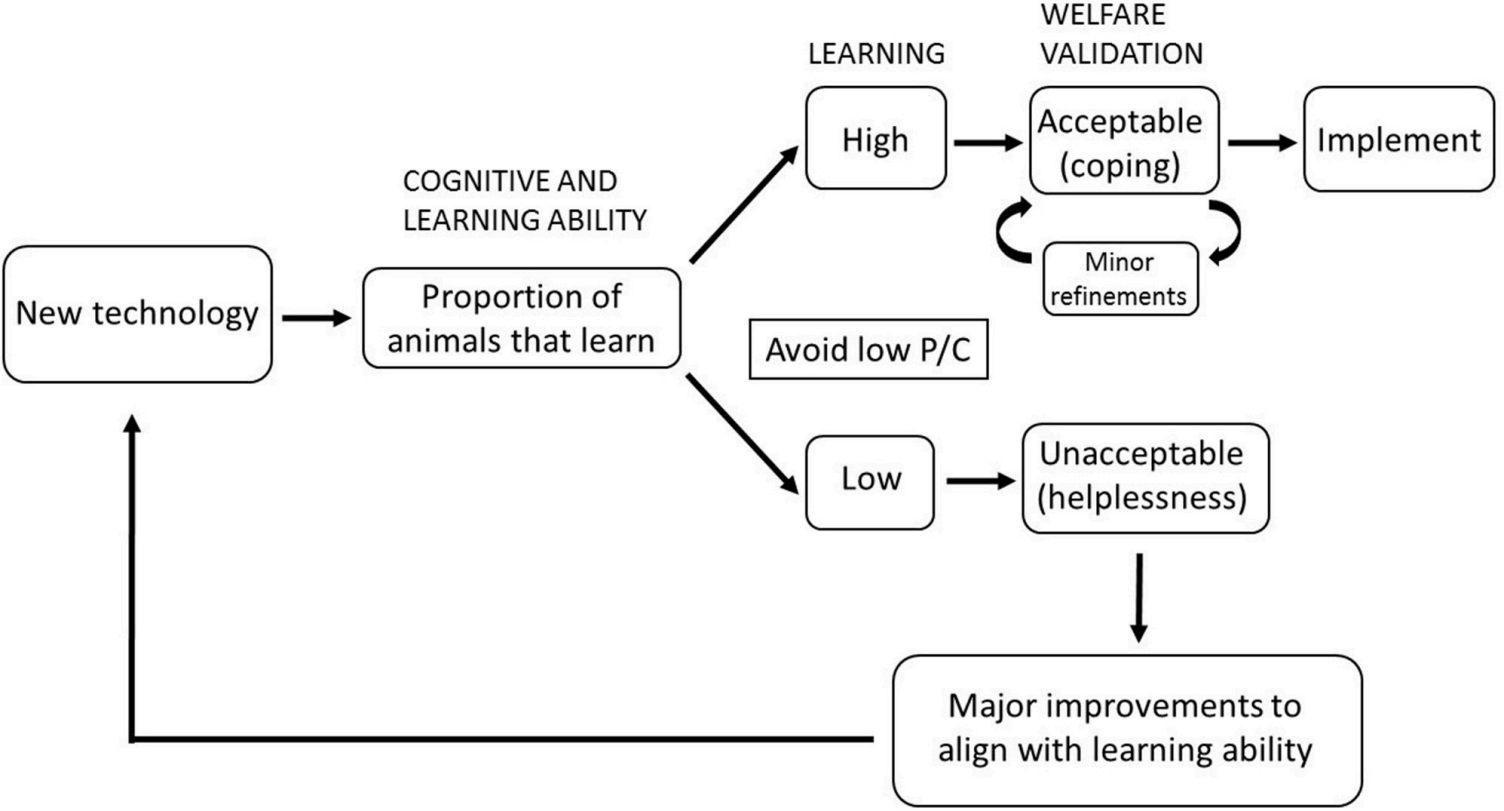
A proposed process to assess welfare outcomes of new technologies or systems applied to livestock. Situations of low Predictability and Controllability (P/C) should be avoided.

To highlight the concepts developed in the framework, a relevant example of a new technology being applied to livestock management is presented. Traditional fences are physical barriers that contain animals by obstructing their passage across a boundary. The traditional barriers can be strengthened by inclusion of aversive stimuli such as spikes (e.g., barbed wire) or electric shocks (e.g., conventional electric fencing). In contrast, virtual fences replace the physical barrier with a benign cue (audio) that heralds the imminent imposition of an aversive stimulus (electric shock) if the animal proceeds across the virtual barrier. Virtual fencing has the potential to reduce labor and material costs associated with moving and maintaining physical fences, enable more efficient pasture management and better protection of environmentally sensitive areas. As the virtual fence is not visible and is more complex for the animal to learn than a conventional electric fence, there may be more interactions with the fence and therefore more shocks received by the animal, however, to date no comparative studies have been reported. For a conventional electric fence, the number of shocks received is highest in the first hour on the first day of exposure (25). With a virtual fence, half the cattle learned to respond to the audio cue after ~6 interactions with the fence (26). Direct contrasts are needed to better understand the comparative impact of the technologies on behavior and welfare. With a commercial system being developed (Agersens®) and a strong demand for the product, it is expected that application of virtual fence technology on farm is imminent. The virtual fencing system utilizes the animal's capacity for avoidance learning through operant conditioning so that the animal learns to respond to an audio cue (conditioned stimulus) to avoid receiving an electric shock (response stimulus). Figure 2 shows the process for assessing welfare outcomes of new technologies such as virtual fencing. With avoidance learning, on the initial approaches to the virtual fence, we suggest that animals are in a situation of low predictability and controllability as they do not know what the audio warning means and are unable to avoid receiving the shock. We interpret this as a negative welfare state as the animals show an acute stress responses to the electric shock (27). After the initial learning period [~6 approaches for 50% of cattle to learn (26)] and with coupling of the application of the audio warning consistently at every approach event, responses indicate that cattle learn the situation has high predictability. If the animal continues to travel further toward the virtual fence line, it receives an aversive electric shock, however, if the animal stops in response to the audio cue and does not proceed across the virtual boundary, it avoids the electric shock. Thus the animal learns to control its exposure to the aversive response stimulus by responding behaviorally to the conditioned audio cue (by turning back from the virtual fence). Avoidance learning by the animal confers high predictability and controllability to the temporal sequence and spatial relationship between the benign conditioned cue and the aversive response stimulus. Through acquisition of the ability to predict and control, the animal's agency improves (28, 29); the resulting welfare state is more positive (Figure 2) and the animal is classified as “coping” (30). Importantly, in avoidance learning, animals that learn and perform well do not display signs of stress, even though avoidance is linked to fear (5).

In contrast, some animals may not have the ability to learn the association between the audio and shock cue, resulting in a situation of low predictability and controllability as described in the studies conducted in rats (5, 7). Whether learning will occur depends on the properties of the events, the consistency of their presentation and the pairing of the stimuli. Ursin and Eriksen (3) termed this predictive value. How an individual perceives the probability of an expected event is termed the perceived probability (31). Predictability is where the perceived probability of stimulus expectancies is high and control occurs when the perceived probability of response expectancies is high (3). As the perceived probability is subjective and dependent on the individual's ability to learn, some animals may not have the ability to learn the appropriate response and therefore the situation for them would be one of low P/C. Individual differences in personality may influence cognitive and learning abilities (32–34). For example, indoor-preferring birds in free range laying systems are more fearful (35, 36) and slower to learn a T-maze test than outdoor-preferring birds (37). More fearful birds use different strategies to learn a task and are less flexible in their learning (38). Further understanding of the relationship between individual differences in personality and cognition will provide guidance in developing operant learning tasks that match learning abilities or for selecting animals better suited to specific production systems. Importantly, for species exhibiting group behaviors such as flocking, motivation to stay close to conspecifics may provide an additional suite of cues to animals that lack an ability to learn the specific conditioned stimulus/responses stimulus paradigm.

## Learning abilities and animal welfare

An acceptable welfare outcome for virtual fencing requires the system to be designed and implemented in a manner that enables animals to learn through prediction and control to avoid the electric shock. While it has been demonstrated in several studies that cattle (26, 39–41) and sheep (42, 43) readily learn to respond to the audio cue, these studies report a large variation between animals in learning speed and task competency. Further research is needed to determine the influence of the duration of training and social environment in which training occurs on learning outcomes. For instance, initial studies suggest that when cattle (26) and sheep (43) were trained individually there was more variation between animals in learning to respond to the audio cue than when trained in groups (41, 42). This is in accord with the potential for social cues to influence learning and task acquisition (44). Further, while acute stress is expected to result when the animals are undergoing avoidance learning, the stress response should be minimal once animals learn to avoid the shock and the situation becomes predictable and controllable.

Animals attempt to learn and adapt to the environment using behavioral and physiological responses, however if the limits to their adaptation or learning ability are reached then chronic stress can occur. Determination of the physiological and behavioral consequences of long-term exposure to virtual fencing in cattle and sheep is needed to ensure that welfare is not compromised. This will include assessment of behavioral patterns, as disturbances in normal time budgets can indicate welfare issues, for example lying time has been demonstrated to indicate comfort of lying surfaces in cattle (45). Over a short-term virtual fencing study in cattle, changes in behavioral time budgets were minor (41), however further research is needed over longer time periods. Learned helplessness may occur if the situation is one of low P/C. Helplessness is associated with chronic stress, and could be assessed for instance through corticotrophin releasing hormone (CRH) or adrenocorticotrophic hormone (ACTH) challenges (13) to monitor welfare impacts of virtual fencing. Increasing complexity of the virtual fencing system such as its use for herding large groups of animals (e.g., mustering dairy cows) and creep feeding young stock will need to be considered in relation to the proposed framework. With increased complexity comes a greater chance of more animals not learning to avoid the shock (i.e., a situation of low predictability and controllability) and a greater potential for poor welfare outcomes. Evidence of animals not coping with a virtual fencing system will require that either the system be altered to ensure learning occurs or that virtual fencing is not implemented for certain groups of animals.

## Conclusions

This paper provides a framework for understanding animal welfare in terms of the animal's affective state and its learned ability to predict and control engagement with its environment. Stress responses occur when animals are unable to predict or control both positive and negative events. This is usually not a welfare issue if the situation of low P/C is short-term, as a normal acute stress response will be observed. However, if the situation is on-going, then chronic stress can be induced, the animal may not be able to cope and welfare outcomes will be poor. There are also potential issues with situations of high and long lasting P/C, which may provide certainty but lack stimulation and lead to boredom. It is recommended that P/C should be intermediate to be of optimal value (5). This intermediate level should be one where the operant task aligns with the learning ability of the animal so that it is predictable and controllable to ensure that welfare is not compromised.

## Ethics statement

This is a review that did not involve any animal experimentation.

## Author contributions

CL conceived the idea and prepared the review. IC contributed to the content, wrote parts, and edited the manuscript. DC contributed to the content, wrote parts, and edited the manuscript.

### Conflict of interest statement

The authors declare that the research was conducted in the absence of any commercial or financial relationships that could be construed as a potential conflict of interest.
